# Improve employee-organization relationships and workplace performance through CSR: Evidence from China

**DOI:** 10.3389/fpsyg.2022.994970

**Published:** 2022-11-03

**Authors:** Yafei Zhang, Chuqing Dong

**Affiliations:** ^1^Research Center of Journalism and Social Development, School of Journalism and Communication, Renmin University of China, Beijing, China; ^2^Advertising and Public Relations Department, Michigan State University, East Lansing, MI, United States

**Keywords:** CSR, employee-organization identification, organizational citizenship behavior, China, survey

## Abstract

Although CSR research in China has received increasing scholarly attention, employee-centered CSR is still an understudied topic. To fill the void, the purpose of this study is to demonstrate the effects of employees’ CSR perceptions on the quality of employee-organization relationships and workplace performance, as well as the underlying mechanisms explaining such effects, in the Chinese context. Guided by both managerial and relational approaches of corporate social responsibility (CSR) research, we conducted a survey (N = 248) with employees from a large private company in the electric and energy industry in China. Data were analyzed *via* path analysis using R packages (e.g., lavaan) and results revealed that CSR perceptions can lead to positive employee-organization relationship quality and increase employees’ extra-role performance. We also found that these effects can be further explained by increased employee-organization identification or perceived corporate ability. In addition, a qualitative analysis of employees’ responses showed that the influence of government and Confucianism was reflected in employees’ expectations for their employer’s CSR initiatives. This study contributes to the scant research on employee outcomes and the underlying mechanisms of employee-centered CSR in China. Practically, we add empirical evidence addressing the value of internal CSR for professionals and educators.

## Introduction

Corporate social responsibility (CSR) can be broadly defined as corporations’ voluntary actions that address social and environmental concerns of stakeholders (Commission of the European Communities, 2002). Although recent years have seen China becoming an important context in international CSR research (e.g., Rim et al., 2019; Zhang et al., 2019; Zhang and Jung, 2020), relatively less is known about how employees perceive and respond to CSR (Zhang et al., 2019). Central to CSR is the relationship management with both diverse stakeholders (Brown and Dacin, 1997; Turker, 2009b; Hofman and Newman, 2014; Story and Castanheira, 2019). However, compared to external CSR stakeholders (e.g., consumers), the internal stakeholders have been significantly understudied in CSR research in general (Gond et al., 2017). Numerous studies have found that employees’ engagement in CSR can lead to positive employee behaviors and organizational outcomes, such as internal organizational culture (Wang and Chaudhri, 2009), job performance (Korschun et al., 2014), organizational identification (Brammer et al., 2015), and organizational citizenship behavior (Lee and Allen, 2002). However, these studies focused primarily on the Western CSR context with little attention to Asia (Kutaula et al., 2020). As Yin and Zhang (2012) suggested, a Chinese perspective is largely missing, despite employees are recognized as the second-largest stakeholder group to Chinese companies (Tang, 2012). Given the unique cultural, political, and institutional contexts of Chinese CSR and the limited scholarly attention to the employee-centered CSR, further research is needed to fill this gap.

Our review of recent studies about employee-centered CSR in China suggests that employees’ CSR perceptions are pertinent to their attitudes and behaviors at the workplace (e.g., Fang et al., 2021; Yan et al., 2021). Despite this growing interest in micro-CSR research, it remains unknown how employees’ CSR perceptions influence both of their quality relationship building and extra-behavioral work. Past research evaluating employee-related CSR outcomes usually focused on behavioral measurements, such as organizational citizenship behavior (OCB; Newman et al., 2016; Paruzel et al., 2021). In contrast, relational outcomes were less studied. As a relational outcome, Employee-organization relationship (EOR) quality is an important indicator that captures employees’ positive relationships with the company. In addition, previous research proposed the typology of CSR and perceived corporate ability (CA) to describe two different types of corporate associations triggering employees’ outcomes (Park and Kim, 2015). However, the relationship between these two associations remains understudied.

The purpose of this study is to explore how employees’ perceptions of CSR influence the quality of EOR and OCB. Drawing from social identity theory, social exchange theory, and existing CSR literature, this study further identified and tested mediating roles of employee-organization identity (EOI) and CA. Moreover, this study analyzed employees’ expectations for their employer’s CSR initiatives, providing qualitative evidence on how Chinese governmental policies and traditional cultural values shape employees’ CSR expectations. A survey was conducted in a leading electric and energy company in China. Data were collected from employees in various positions, including both managerial and frontline employees across different departments (e.g., marketing and R&D departments). After data cleaning, 248 valid responses were used to test the proposed relationships *via* path analysis in relevant R packages (e.g., lavaan).

This study contributes to the current CSR research by addressing CSR effects from the employee perspective and adding empirical evidence from a developing country where CSR is fast growing. Specifically, this study introduced a relational outcome (EOR) and a potential mediating mechanism (CA) through which employees’ CSR perceptions could affect employees’ relations and behaviors towards their companies. Our study also extends the existing literature by incorporating a qualitative analysis of employees’ expectations in their companies’ CSR initiatives. Practically, our findings provide meaningful guidance for managers in Chinese firms, especially those in the energy industry, to understand the value and effects of their CSR efforts.

The rest of the paper is structured as follows. The paper starts with a review of CSR in China highlighting the unique context and the current CSR research themes in China. Then, hypotheses are proposed based on the social identity theory, social exchange theory, and the literature of key variables. The method section includes the procedure, measures and the analysis approach. Then, the results are presented, followed by the discussion, theoretical and practical contributions, and limitations and future research.

## Literature review

### CSR in China

The extant CSR research in the Chinese context mainly focuses on the following themes, including (1) drivers for CSR performance, (2) CSR information disclosure, and (3) CSR practices and the impact on the corporate outcomes, and (4) CSR and its stakeholders.

First, there is proliferate literature exploring factors affecting CSR performance in China. Compared to a set of dominant market drivers (e.g., consumers, employees, investors, and business suppliers) and social drivers (e.g., NGOs and media) in the CSR development of Western countries, CSR in China involves many other essential drivers (Zhang et al., 2019), such as the Confucianism culture, government, and economic development. The essence of Confucianism includes *ren* (compassion and benevolence), *yi* (moral righteousness), *li* (principles and norms), *zhi* (wisdom), *xin* (trustworthiness; Wang and Juslin, 2009). Therefore, according to those Confucian values, business merchants are encouraged to bring more societal and individual benefits beyond profit-making (Zhao et al., 2019). The government plays a “directing role in promoting and implementing CSR in China” (Tang et al., 2018). The unique political context makes CSR development in China a “top-down” approach since the governmental policies, regulations, and laws guide companies to plan their CSR initiatives (Tang et al., 2018). In addition, institutional factors such as the ownership of the company and the regional development can affect Chinese companies’ CSR decisions (Ali et al., 2019). The rising Chinese domestic consumers’ CSR awareness, the rapid economic development, and multinational companies’ global outreach to China also enhance the CSR development in China (Zhang et al., 2019; Wang et al., 2020).

Second, an increasing number of studies examined how Chinese corporations disclosed CSR information. For instance, Rim et al. (2019) examined CSR reports of large corporations in U.S., South Korea, UAE, and China and found that compared to companies in other countries, Chinese companies disclosed more substantial information in CSR reporting, whereas showed less accountability and stakeholder participation in their CSR reporting. Industry characteristics also influence Chinese companies’ CSR reporting. Specifically, Rim et al. (2019) found that Chinese companies in the environmentally sensitive industries were more responsive to stakeholder pressures on corporate environmental performance, resulting in more transparent communication in CSR reports. Hu et al. (2020) suggested that CSR information disclosure can make investors more informed, contributing to the investment in innovation sustainability.

The third line of CSR research in China highlighted how CSR practices could influence corporate outcomes. For instance, Ali et al. (2019) concluded that companies’ socially responsible performance was positively associated with their financial performance. Wu et al. (2020) examined the CSR index and corporate financial performance index in the Chinese manufacturing industry and suggested a positive correlation between these two indexes. Zhang and Jung (2020) conducted a longitudinal study and suggested that CSR had a significant positive influence on corporate value from 2013 to 2018. The last theme of CSR research was about how CSR engaged with a variety of stakeholders in China. The majority of research in this line focused on external stakeholders, such as consumers. For example, Wang et al. (2020) employed an experiment to explore how green information affected consumers’ purchase intention and suggested that consumers had a higher purchase intention when they obtained green information from the company. Kim (2022) surveyed consumers in Mainland China and Hong Kong and concluded that the consumers’ perceived CSR communication was positively associated with their CSR knowledge, CSR engagement and trust in CSR commitment, which in turn, boosted their perceptions of corporate reputation.

The extensive literature review of CSR research in China revealed that most research has overwhelmingly focused on external stakeholders; relatively less attention has been given to how employees perceive CSR and the consequential organizational outcomes and employee behaviors. Among a few empirical studies, Newman et al. (2015) suggested that employees’ perceived CSR importance was positively associated with their job performance and OCB. Zhao et al. (2019) surveyed Chinese employees and indicated that employees’ CSR perceptions were positively associated with their organizational identification. Fang et al. (2021) surveyed employees across different industries in China and concluded that employees’ CSR perceptions were positively associated with their job performance and such effect was mediated by industrial relations climate and psychological contract fulfillment. Yan et al. (2021) found that employees’ CSR perceptions were positively associated with employees’ behaviors to challenge the company’s status quo. However, these studies did not provide clear and consistent explanations on the mechanisms through which employees’ CSR perceptions exerted an impact on their behavioral and relational outcomes concerning their company. As Gond et al. (2017) suggested, more research on employee-centered CSR is needed.

Organizations today are highly expected to be socially responsible for a variety of stakeholders (Kim, 2019). Different from employees’ CSR perceptions, employees’ CSR expectations highlight what CSR focuses that they hope their employers will engage with. Kim (2019) suggested that consumers’ CSR expectations (e.g., informativeness and transparency) were positively related to their corporate reputation perception. In a similar rationale, employees’ CSR expectations could matter to their overall engagement with the company. Given the scant literature on employees’ expectations for CSR, this study proposes the exploratory question in the Chinese context:

RQ: What CSR do employees expect the most from their employer?

### Employee perceptions of CSR

CSR has received interdisciplinary research interests from economics, marketing, management, and communication fields. However, previous research has not reached a universal definition of CSR (Visser, 2006; Carroll, 2008). In addition, similar concepts such as corporate citizenship and sustainability also enrich the CSR concept (e.g., Capriotti and Moreno, 2007). A widely acknowledged CSR conceptualization was proposed by Carroll (1991), suggesting that CSR is multidimensional and can be understood using a pyramid structure. This CSR conceptualization stated that corporations should meet four layers of societal expectations toward CSR initiatives: economic, legal, ethical and philanthropic responsibilities (Carroll, 1991, 1999). However, some scholars (e.g., Turker, 2009b; Aguinis, 2011) have argued that producing goods and services for society is a fundamental function of business, and legality is different from CSR. In addition, most researchers in CSR now highlight CSR practices that are beyond the company’s profitable interests and legal requirements (McWilliams and Siegel, 2001; Brammer et al., 2015).

Following their arguments, this study defines employee perceptions of CSR activities as the extent to which employees perceive a company’s behaviors that “aim to affect stakeholders positively and that go beyond its economic interest” (Turker, 2009b, p. 413–414). More specifically, CSR perception is categorized into society, employees, customers and government dimensions from employees’ perspectives (Turker, 2009a). CSR towards society indicates that companies contribute to social welfare and development. CSR towards employees refers to companies’ support and responsibilities in protecting internal stakeholders’ benefits, such as career opportunities and job security. CSR towards customers represents companies’ responsibility towards their consumers, products, and services. CSR towards the government indicates that companies comply with relevant laws and governmental regulations and pay taxes. Turker’s conceptualization of employees’ CSR perceptions was widely applied in empirical CSR research. For instance, Fang et al. (2021) found that employees’ CSR perceptions towards multi-stakeholders (i.e., employees, consumers, government, society) significantly influenced their job performance in China. Castro-González et al. (2021) contextualized the conceptualization in Spain, and suggested that salespeople’s CSR perceptions reduced their turnover rate.

Rupp et al. (2006) suggested that employees form three types of judgements regarding their employer’s socially responsible actions: (1) social concerns addressed by organizational actions, (2) the outcomes of such actions, and (3) how stakeholders, both inside and outside the organization, are affected by such actions. Thus, employees’ overall perception of a firm’s CSR is multidimensional and inclusive (Rupp et al., 2006; Dhanesh, 2014), rather than simply focusing on employee-related CSR programs. Given that CSR is a growing phenomenon in China (Moon and Shen, 2010) and has not been clearly defined, this study uses inclusive CSR perceptions concerning a broad scope of CSR from the consumer, employee, government, and social aspects. In a recent meta-analysis, Paruzel et al. (2021) suggested that employees’ CSR perceptions showed positive effects on individuals’ attitudes and behaviors toward their companies. The following sections will specifically introduce OCB as an employee behavioral outcome and EOR as a relational outcome regarding the effects of employees’ CSR perceptions in this study.

### Employee organizational citizenship behavior

This study posits that organizational citizenship behavior (OCB) is a key behavioral reaction of employees to a firm’s CSR activities. OCB is an important indicator of workplace performance from an organizational perspective. OCB has been defined in various ways in the literature (e.g., Borman and Motowidlo, 1993). In general, OCB refers to “employee behaviors that, although not critical to the task or job, serve to facilitate organizational functioning” (Lee and Allen, 2002, p. 132). OCB is different from job performance given its focus on extra-role behaviors rather than job requirements (Lee and Allen, 2002). Extra-role behaviors are employee-initiated activities that are over and above the scope of job descriptions, which helps their organization to further succeed (Niehoff and Moorman, 1993; Lee and Allen, 2002; Newman et al., 2015). These behaviors include, but are not limited to, helping colleagues, attending functional meetings that are optional (Lee and Allen, 2002), and creating positive word-of-mouth messages to outsiders (Podsakoff et al., 2000).

CSR perceptions have empirically been shown to generate beneficial organizational outcomes such as image building, reputation enhancement, and increased purchase intention. While these areas have been researched extensively (Du et al., 2010), only in recent years has research begun to examine workplace performance as an indicator of CSR effectiveness (Newman et al., 2015). Compared to external stakeholders, employees may have a stronger awareness of their firm’s CSR efforts. Emerging research has examined how employees’ different CSR-related perceptions influence their OCB, suggesting that employees’ perceptions of CSR related to community relations and environmental sustainability have a positive influence on OCB (Rupp et al., 2013). Jones (2010) focused on employees’ evaluations of their companies’ volunteer work and found a positive relation to OCB. Zhang et al. (2014) found that CSR perceptions towards internal stakeholders (i.e., employees) were positively related to OCB. Newman et al. (2015) also found that employee perceptions of CSR targeting secondary stakeholders, such as NPOs and communities, significantly affected OCB. In line with these findings, this study argues that employees’ CSR perceptions have a positive impact on OCB.

*H1a*: Employees’ perceptions of organizational CSR will be positively related to their OCB.

### Employee-organization relationships

EOR considers relationship quality a key indicator of successful public relations practices. In addition to the employee behavioral outcome, namely OCB, this study treats EOR as a relational outcome of CSR communication, which is a specific type of organization-public relationships focusing on internal relationship management (Jiang, 2011). According to the previous literature (Hon and Grunig, 1999; Huang, 2001; Jiang, 2011), the quality of EOR can be indicated by employee perceptions of trust, satisfaction, and commitment (e.g., Turker, 2009a; Farooq et al., 2014). Trust indicates an employee’s level of confidence in and willingness to be open to his/her company (Hon and Grunig, 1999). Specifically, it includes the employee’s beliefs about the company’s integrity, dependability, and competence (Hon and Grunig, 1999). Satisfaction refers to the extent to which an employee favors his/her company, which highlights the affective and emotional feelings towards the company (Kang and Sung, 2017). Commitment indicates the cognitive and affective beliefs held by both parties regarding the worthiness of maintaining and promoting the current relationships (Hon and Grunig, 1999).

According to social exchange theory, trust, satisfaction, and commitment between employees and the organization are likely to be reciprocal (Gergen, 1969). If employees recognize that the company’s CSR efforts benefit them as well as other related stakeholders, the employees will feel obligated to reciprocate the favor (Jones, 2010; Farooq et al., 2014). Previous research has indicated that various perceptions of CSR lead to different levels of trust toward, commitment to, and satisfaction of the employer. West et al. (2015) examined employee-perceived CSR in a Canadian retailer and suggested a positive relationship with employee trust towards the firm. Peterson (2004) also suggested that perceived CSR could affect employees’ commitment to the organization since employees would feel proud to identify with a socially responsible organization and it would boost their self-esteem. Turker (2009b) found that employees’ CSR perceptions of society, employees, customers, and government were positively related to organizational commitment. In the Chinese context, Hofman and Newman (2014) surveyed 280 employees in Chinese companies and concluded that employees’ CSR perceptions of internal stakeholders were positively related to their organizational commitment. Thus, this study hypothesizes that CSR would cultivate EOR.

*H1b*: Employees’ perceptions of organizational CSR will be positively related to EOR.

### Employee-organization identification

Social identity theory (Tajfel and Tumer, 1985) suggests that people go beyond their personal identity to develop a social identity in order to articulate their sense of self. They do so by identifying themselves as members of various social categories with which they have a collective identity. Employee-organization identification is a “specific type of social identification” (Ashforth and Mael, 1989, p. 22), providing a basis for individual employees’ loyalty and commitment to the organization (Ashforth and Mael, 1989). Through EOI, employees establish cognitive connections with the organization, which facilitates the process of internalizing the organizational values and beliefs (Ashforth and Mael, 1989; Stuart, 2002). Employees who strongly identify with their organizations are more likely to participate the organizational functions as their own (Dutton et al., 1994) and to be involved in activities that reinforce the organizational goal achievement (e.g., van Dick et al., 2007). For instance, if an employee claims to be a member of an organization that is socially responsible, such identification would make the employee think and behave in a similar way to that of the organization (Newman et al., 2015).

In public relations research, identification is considered an antecedent of positive EOR, mostly for external stakeholders. Consumers want to identify with the company when the company links itself to socially responsible behaviors (David et al., 2005). Consumer-company identification also contributes to positive attitudes towards the company (Brown and Dacin, 1997), increasing loyalty (Bhattacharya and Sen, 2003), positive word-of-mouth intentions (Bhattacharya and Sen, 2003; Hong and Yang, 2009), and stronger purchase intentions (Bhattacharya and Sen, 2003; David et al., 2005; Kim, 2017). In addition, David et al. (2005) suggested that consumers’ perceptions of a company’s ethical CSR practices influence their consumer-company identification in a positive way, and their perceptions of CSR relational practices positively relate to consumer-company identification leading to greater purchase intention. Similarly, Kim (2017) concluded that consumers were more likely to purchase products/services when they perceived that the company proactively conducted socially responsible behaviors.

Given the relationship between identification with the company and relational outcomes, employees who are well-informed about the company’s CSR initiatives would develop more relational trust, satisfaction, and commitment in EOR. These previous findings lead us to the following hypotheses:

*H1c*: Employees’ perceptions of organizational CSR are positively related to EOI.

*H2a*: EOI is positively related to OCB.

*H2b*: EOI is positively related to EOR.

*H4a*: EOI mediates the relationship between employee perceptions of organizational CSR and OCB.

*H5a*: EOI mediates the relationship between employee perceptions of organizational CSR and EOR.

### Perceived corporate ability

Corporate ability is defined as “a company’s expertise in producing and delivering its outputs” (Brown and Dacin, 1997, p. 68). Both CSR and CA were identified as two types of corporate associations (Brown and Dacin, 1997). A number of studies have examined the role of CA from consumers’ perspectives, including the relationship between perceptions of product/service quality, favorable attitudes (Folkes and Kamins, 1999), purchase intention, and customer loyalty (Sen and Bhattacharya, 2001; David et al., 2005). CSR perceptions and perceived CA are two important factors in predicting people’s evaluations of the company and relevant products/services (Kim, 2011, 2014; Xie and Peng, 2011; Park and Kim, 2015). Park and Kim (2015) found that consumers’ perceptions of CSR offered a larger buffering effect than perceived CA in corporate crisis issues. Consumers who had a positive attitude towards the company before a crisis were less likely to blame it on internal causes. Kim (2014) further examined how consumers’ prior CSR and CA perceptions affected their attitudes towards the company related to product-crisis issues. The author found that consumers’ positive prior CSR perceptions positively influenced the company more than their perceived CA in a product-crisis issue. Xie and Peng (2011) suggested that both perceived CSR and CA were positively related to competence-based trust and identity-based trust, which consequently contributed to a strengthened individual-company relationship.

Taken together, these studies have provided evidence that CSR and CA have a joint influence on the public’s evaluation of a firm. Nevertheless, most previous research has mainly focused on the comparative effects of CSR perceptions and perceived CA. Among the few studies that have examined the relationship between perceived CSR and CA, Park and Kim (2015) indicated that consumers still showed supportive attitudes towards companies with good CSR performance even if they perceived that the company’s CA was poor. Kim (2014) tested the relationship between CSR and CA and concluded that positive prior CSR activities were more effective than positive CA in decreasing consumers’ dissatisfactions in product-crises. In terms of the effects of CA, Brammer et al. (2015) examined CA as a moderator between employees’ reactions to CSR and their identification with the organization and concluded that when employees perceived CA as higher than CSR, the employees’ organizational identification was strengthened. In addition, employees may be proud to be connected to a capable organization, and CA helps employees develop trust and security in the organization (Brammer et al., 2015). As a result, positive CA could contribute to better job performance for employees. Thus, we develop the following hypotheses:

*H1d*: Employees’ perceptions of organizational CSR are positively related to perceived CA.

*H3a*: Perceived CA is positively related to OCB.

*H3b*: Perceived CA is positively related to EOR.

*H4b*: Perceived CA mediates the relationship between employee perceptions of organizational CSR and OCB.

*H5b*: Perceived CA mediates the relationship between employee perceptions of organizational CSR and EOR.

## Materials and methods

### Data collection and sample

Data were collected from a large private company in China in the electric and energy industry. The electric and energy industry is committed to energy generation and transmission, which provides solutions to businesses to save energy and protect the environment. In the past decade, China has been criticized for its overemphasis on economic development and lack of focus on sustainability and energy optimization (Xu and Yang, 2010). We selected the electric and energy industry because the CSR practices are highly visible and this industry is facing growing challenges related to resource scarcity, climate change, and environmental pollution (Stjepcevic and Siksnelyte, 2017). This study focused on one single industry to avoid potential confounding effects from various industries (Eisenhardt, 1989; Yin and Zhang, 2012).

The selected company is a leading electric company in China that is committed to energy-saving solutions for a variety of sectors, including cement, wood, sewage, and chemical engineering. The selected electric company has also engaged in CSR practices, including philanthropy, community involvement, and fair employee treatment. In addition, the selected company is one of the largest employers in the industry.

The data were collected from employees in various positions, including lower-level employees and lower-and middle-level managers from several departments including production, marketing, research and development, and administration. Participants were asked to complete a translated (from English to Chinese) questionnaire during their work hours on the company premises. We adopted the translation-back translation approach to ensure face validity. The translated questionnaire was also proofread by educated Chinese professionals with Chinese-English translation certificates. Data from 248 participants were used for analysis, among which 58.1% were males (*n* = 144) and 41.9% were females (*n* = 104). The average age was 28.9 years old (*SD* = 14.12). The average working experience in the company was 3.0 years (*SD* = 1.62).

After reading the consent form, participants were presented with a brief statement describing the definition of CSR. Participants were told that their participation was voluntary and the survey was for research purposes only. The survey was anonymous and all responses were kept confidential by the research team. After they consented to participate, they were asked to describe their perceptions of the CSR practices of the company, employee-company identification, corporate ability, and relational and behavioral outcomes based on their own experience. After the close-ended questions, at the end of the survey, participants were asked to write down what CSR activities they think their company should initiate/engage with.

### Measures

The key variables in this study were all adapted from previous literature. *Employee CSR perceptions*: 11 items were adapted and developed from Turker (2009b), including society, employee, consumer and government perspectives (*M* = 5.25, *SD* = 1.48, Cronbach’s *α* = 0.93). Sample items are, “The company participates in activities that aim to protect the quality of the environment” (society), “The company is primarily concerned with employees’ needs and wants” (employee), “The company respects consumer rights beyond the legal requirement” (consumer), and “The company always pays its taxes on a regular and continuing basis” (government). All items were rated on a 7-point Likert scale from *never* (1) to *always* (7).

*EOI*: three items were modified from Dutton et al. (1994); *M* = 5.38, *SD* = 1.28, Cronbach’s *α* = 0.92). A sample item is, “I am part of my company.” All items were rated on a 7-point Likert scale from *strongly disagree* (1) to *strongly agree* (7).

CA: CA was measured by six items adapted from previous literature (*M* = 5.26, *SD* = 1.40, Cronbach’s *α* = 0.94; Brown and Dacin, 1997; Kim and Rader, 2010). A sample item is, “I associate the company with good quality products.” All survey items were rated on a 7-point Likert scale from *strongly disagree* (1) to *strongly agree* (7).

*EOR*: A 14-item scale adapted from Huang (2001) was used to measure EOR, including trust, relationship satisfaction, and relationship commitment (*M* = 5.05, *SD* = 1.51, Cronbach’s *α* = 0.98). Sample items are, “The company can be relied on to keep its promises” (trust), “I am happy with the company” (relationship satisfaction), and “There is a long-lasting bond between the company and people like me” (relationship commitment). All survey items were rated on a 7-point Likert scale from *strongly disagree* (1) to *strongly agree* (7).

*OCB*: OCB was measured by five items from Lee and Allen (2002; *M* = 5.42, *SD* = 1.32, Cronbach’s *α* = 0.94). A sample item is, “I keep up with developments in the company.” All survey items were rated on a 7-point Likert scale from *strongly disagree* (1) to *strongly agree* (7).

### Data analysis

To test hypotheses, path analysis techniques were used to examine the direct and indirect effects from employee CSR perception to the relational (EOR) and behavioral outcomes (OCB) *via* EOI and perceived CA, respectively. Demographic variables (gender, age, and working experience) were treated as control variables in the model. The path analysis was conducted by the lavaan R package. To answer the research question, the responses of the open-ended question were analyzed by textual analysis techniques following the protocol of saturation and commonality in the data (Guest et al., 2011; Lindlof and Taylor, 2017).

## Results

Prior to the analysis, we conducted a multivariate normality test of all endogenous variables (EOI, CA, OCB, and EOR) using the Shapiro–Wilk test, and *p* values were all smaller than 0.5, which indicated the violation of multivariate normality. Thus, we used the “robust standard errors to analyze the data with a normal theory method” (Kline, 2011 p.177). In particular, we used Satorra-Bentler corrections (MLM) instead of maximum likelihood (ML) as the estimator in the path analysis to test our hypotheses. The correlation matrix of endogenous and exogenous variables was displayed in Table 1.

**Table 1:** The correlation matrix of variables.

Constructs	Employee perceived CSR	EOI	CA	EOR
EOI	0.71			
CA	0.71	0.73		
EOR	0.80	0.76	0.79	
OCB	0.73	0.74	0.63	0.81

H1 posited that employee CSR perception was positively associated with OCB (H1a), EOR (H1b), EOI (H1c), and perceived CA (H1d). As indicated in Table 2, our results suggested that employee CSR perception was positively associated with OCB (*β* = 0.38, *p < 0.001*), EOR (*β* = 0.41, *p < 0*.001), EOI (*β* = 0.72, *p < 0*.001), and CA (*β* = 0.73, *p < 0*.001). Therefore, H1a, H1b, H1c, and H1d were all supported.

**Table 2:** Results of direct effects.

Hypothesis	Unstandardized estimate	SE	Standardized estimate	Test result
Employee CSR perceptions -> OCB	0.38	0.10	0.38***	Supported
Employee CSR perceptions -> EOR	0.48	0.10	0.41***	Supported
Employee CSR perceptions -> EOI	0.81	0.05	0.72***	Supported
Employee CSR perceptions -> CA	0.80	0.05	0.73***	Supported
EOI -> OCB	0.35	0.09	0.40***	Supported
EOI -> EOR	0.23	0.10	0.23*	Supported
CA -> OCB	0.10	0.08	0.11	Not supported
CA -> EOR	0.34	0.09	0.32***	Supported

N = 248.

* *p* < 0.05;

*** *p* < 0.001.

H2 predicted that EOI was positively associated with OCB (H2a) and EOR (H2b). As seen in Table 2, our results supported the positive relationship between EOI and OCB (H2a; *β* = 0.40, *p < 0*.001), and the positive relationship between EOI and EOR (H2b;(*β* = 0.23, *p < 0*.05). Therefore, both H2a and H2b were supported.

H3 concerned the positive impact of perceived CA on OCB (H3a), and on EOR (H3b). The results, however, did not support the positive impact of perceived CA on OCB (H3a; *β* = 0.11, *p = n.s*). In contrast, perceived CA was significantly associated with EOR in a positive way (H3b; *β* = 0.32, *p < 0*.001). Therefore, only H3b was supported.

H4 tested the mediating roles of EOI (H4a) and perceived CA (H4b). Table 3 showed that employee CSR perception exerted a direct positive effect on OCB (*β* = 0.38, *p <* 0.001), and it also indirectly influenced OCB only through EOI (*β* = 0.29, *p < 0.001*) with a total effect of.75 (*p < 0*.001). In contrast, perceived CA did not mediate the effect of employee CSR perception on OCB as the indirect effect was not found (*β* = 0.08, *p* = n.s.). Therefore, H4a was supported, but H4b was not supported.

**Table 3:** Standardized direct and indirect effects of employee CSR perceptions on OCB.

	Direct effects	Indirect effects	Total effects
Employee CSR perceptions	0.38***	0.37***	0.75***
EOI	0.40***	0.29***	
CA	0.11	0.08	

*N* = 248.

*** *p* < 0.001.

**Figure 1:**
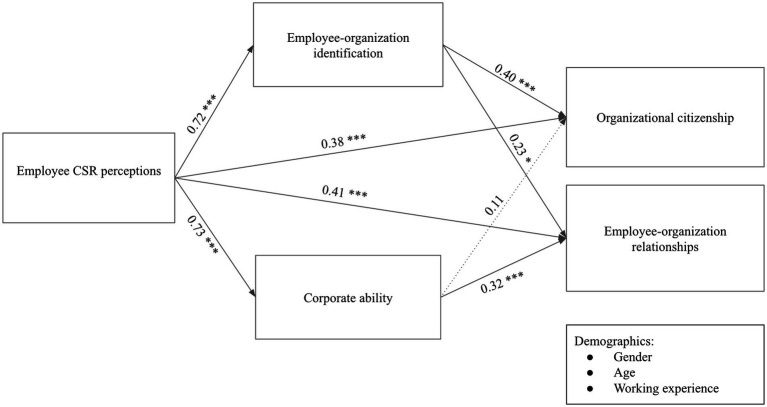
Estimated standardized effects in path analysis.

H5 examined the mediating effects of EOI (H5a) and perceived CA (H5b). Table 4 showed that employee CSR perception had a direct positive effect on EOR (*β* = 0.41, *p* < 0.001) and it indirectly influenced EOR through both EOI (*β* = 0.16, *p* < 0.001) and CA (*β* = 0.23, *p* < 0.001), respectively, with a total effect of.80 (*p* < 0.001). Therefore, both H5a and H5b were supported. Results were also displayed in Figure 1.

**Table 4:** Standardized direct and indirect effects of employee CSR perceptions on EOR.

	Direct effects	Indirect effects	Total effects
Employee CSR perceptions	0.41***	0.39***	0.80***
EOI	0.22***	0.16***	
CA	0.32***	0.23***	

*N* = 248.

*** *p* < 0.001.

As for the research question on which CSR activities the employer should engage in, 50.81% (*n* = 126) out of 248 participants answered the question, and answers can be categorized into the following themes. First, the majority of employees (45.23%, *n* = 57) identified that their employer should mostly pay attention to societal and environmental issues, including poverty alleviation, charity, volunteer work, NGOs, more available job positions, environmental protection, and support for equal education opportunities. Second, employees indicated that their company should mostly participate in CSR activities that can benefit employees (27.78%, *n* = 35), including employee well-being, fair treatment at the workplace, a balance between work and life, an increase in salary, and higher employee retention. Third, some employees (9.52%, *n* = 12) recognized R&D development and product improvement as the most salient CSR activity that the company should pay attention to. Fourth, fewer employees prioritized CSR focus on the government (3.17%, *n* = 4), including paying tax and abiding by laws. Last, a handful of employees (7.94%, *n* = 10) suggested that the company’s CSR main focus should be on Chinese cultural values, including honesty and trustworthiness, social and business virtues.

## Discussion

Guided by both managerial and relational approaches of CSR research, this study empirically tested a model to understand the effects of CSR perceptions on the quality of employee-organization relationships and workplace performance in a Chinese business context. The study’s results supported that employees’ CSR perceptions have direct positive effects on OCB, EOR, EOI and CA. Given the understudied mechanisms linking employees’ CSR perceptions and employee outcomes (Glavas, 2016; Wang et al., 2020), the findings highlighted the mediating roles of EOI in the relationships between employee CSR perception and relational (i.e., EOR) and behavioral outcomes (i.e., OCB), respectively. Our findings fill gaps in the current CSR research by highlighting employee-relevant outcomes as important indicators of CSR effectiveness and add empirical evidence from a non-Western perspective.

To elaborate, this study first confirmed the positive relationships between employee perceived CSR and OCB. The findings indicate that firms’ involvement with a diverse scope of CSR initiatives can be rewarding because when more CSR practices are perceived, employees are more likely to work beyond their assigned job tasks to benefit their organizations. Basu and Palazzo (2008) suggested that CSR can be conceptualized as employees’ sense-making process, and positive CSR perceptions can enhance their pro-organization behaviors (Edinger-Schons et al., 2019). Our findings supported this internalization process by empirically showing the connections between workplace behaviors and corporate CSR behaviors. CSR suggests a guideline of OCB and employees would like to participate in the company’s CSR initiatives by showing OCB (Paruzel et al., 2021). In addition, OCB includes a positive word-of-mouth communication about the employer. The positive impact of an employer’s CSR actions on employees is likely to have a broader social impact throughout employees’ individual networks, consequently influencing other external publics’ perceptions of the firm. For example, employees could be CSR ambassadors of their companies, and communicate the underlying values of the company to external stakeholders, including consumers (Korschun et al., 2014; Edinger-Schons et al., 2019). Corporate CSR initiatives may, therefore, create an “inside-out” positive effect starting with the positive influences on employee behaviors (Morsing et al., 2008).

Our study found that employees’ perceptions of CSR positively influenced EOR, which is consistent with previous research (e.g., Dhanesh, 2014; West et al., 2015), suggesting that CSR may serve as an EOR relationship cultivation strategy (Dhanesh, 2014). Employees with positive perceptions, (i.e., the more corporate CSR initiatives that are perceived by the employees) were more likely to engender higher trust, commitment, and satisfaction toward their employer. Following the logic of social exchange (Hofman and Newman, 2014), employees are more likely to reciprocate and maintain a high-quality relationship with a company that is favorable to CSR practices. Previous research has found that most Chinese companies equated CSR only with charitable behaviors (Wang and Chaudhri, 2009). In this study, participants responded to various dimensions of CSR practices, and focused on both external (e.g., society) and internal (e.g., employee) stakeholders. Thus, the findings suggested that employees’ overall perception of CSR is important, which challenges corporations’ narrow understanding of CSR. Thus, the company’s communication about CSR activities to employees should include diverse aspects of CSR related to consumers, employees, government, and society.

This study suggested a positive relationship between how employees perceived their company’s CSR activities and their identification with the organization, which provides additional evidence that highlights the role of identification in positive employee behaviors (Farooq et al., 2014; Newman et al., 2015). The increased identification with the organization could be explained by the increased feeling of pride employees experienced after observing the firm’s CSR initiatives. Jones (2010) also examined employee perceptions of organizational volunteer work towards external stakeholders and suggested that employees valued volunteer practices of their employer and would identify with it because the organization “makes them feel prouder about their organizational membership” (Jones, 2010, p. 870).

Our results also indicated that employee CSR perceptions positively influence employee’s identification with the organization, which is ultimately linked to the increased OCB and better EOR. Therefore, employee-organization identification could be considered as an underlying mechanism explaining the relationships between employee CSR perceptions and OCB and EOR. Social identity theory was the most prominent theory to explain the effect of CSR on employee outcomes (Paruzel et al., 2021). Our findings of EOI as a mediator in the Chinese context further substantiate the solid underlying mechanism of EOI between CSR and OCB (Newman et al., 2016; Farooq et al., 2017). OCB is not mandated under employees’ required job fulfillment. When employees find their companies’ CSR initiatives align with their prosocial values, they would be more likely to feel attached and identify with their companies. As a result, such positive feelings could further motivate employees to perform beyond their job duties.

EOR is a long-term measure of the relationship quality and may need repeated observations and experiences (Hon and Grunig, 1999). Our findings echo previous literature suggesting that CSR enhances consumer-company identification and consequently improves consumers’ long-term relationship with the company (Bhattacharya and Sen, 2003; Du et al., 2007; Hong and Yang, 2009). Compared to consumers, employees have more complicated relationships with a firm such that they can be observers, participants, planners, and/or benefactors of CSR practices. In addition, employees experience power dynamics in relation to their company. Therefore, it is more important for employees to develop a sense of belonging with the company. Companies’ CSR engagement is one of the most prestigious social attributes (Paruzel et al., 2021), and it can significantly enhance employees’ identification and belongingness with the company, which consequently builds the long-term trust of, satisfaction with, and commitment towards the company.

Another interesting finding is the positive association between employee-perceived CSR and CA. Unlike previous research treating CSR associations and CA associations as parallel constructs, this study attempted to link the perceived CSR and CA and revealed a positive spillover effect between the two variables. Our findings somewhat echo Kim’s (2011) argument that there is a potential transfer effect from CSR to CA in her synergistic model of corporate communication strategy. In a highly competitive market, companies need to meet both their economic bottom line and the demand for social responsibility. Although CSR and CA represent two distinct corporate characteristics, our findings suggest that they are not separate entities. Rather, they are integrated, especially from an employee’s standpoint. In addition, perceived CA also mediates the positive relationship between employee CSR perceptions and EOR. Employees who perceive their companies perform well in CSR will have an improved feeling of corporate’s overall ability, and the spillover effect can enhance employees’ psychological attachment (e.g., commitment and trust) with the company. That means the reward of a greater perception of CSR on EOR lies in the increased perceived corporate ability.

However, our findings did not find any direct and indirect effects between employee CSR perceptions and OCB *via* CA. Although CA could help employees build trust and security with the organization (Brammer et al., 2015), it might also reduce employees’ motivation as it could violate the perceived personal efficacy and usefulness (Ryan and Deci, 2000). In other words, employees may perceive that their extra-role behaviors make less of a difference to the organization. OCB involves employees’ voluntary actions that benefit others and the firm. If employees believe that the firm is capable enough, they might hold back on duties beyond their job requirements.

Employees’ expectations for CSR reflect the unique social and political CSR context in China. A salient theme in our finding is that employees expect their employer to take targeted measures to alleviate poverty, provide more job positions, and support equal education opportunities. The qualitative finding is consistent with the existing CSR research in China highlighting unique institutional factors affecting CSR practices in the Chinese context (Ali et al., 2019). Specifically, the finding echoes the government’s leading role in CSR promotion and implementation in China. For example, the Chinese government encouraged state-owned enterprises and the private sector to cooperate with the local government to merge resources and take targeted measures to alleviate poverty. Companies owned by the government (state) outperformed other ownerships of companies in CSR practices in China (Ali et al., 2019). As a result, employees were aware of these state-led CSR policies and expected the company to take a proactive approach in integrating them into CSR initiatives. In addition, our finding also revealed that employees relied on Chinese cultural values, such as honesty and trustworthiness in describing their expectations for the company’s CSR. The finding was consistent with previous research in CSR in China highlighting the unique cultural context (e.g., Zhang et al., 2019; Zhao et al., 2019). Honesty and trustworthiness were two core values in Confucianism, and these cultural values are pertinent to employees’ understanding of salient CSR initiatives in contemporary China.

We concluded this article by providing the theoretical and practical implications, discussing current limitations, and considering future research to enrich the scholarship in employee-centered CSR.

### Theoretical and practical implications

This study makes several contributions to the Chinese CSR scholarship. Specifically, consistent with recent studies (e.g., Lim and Jiang, 2021), our study suggests that CSR can effectively cultivate relationships between the organization and its employees in the Chinese context. Our study emphasizes the importance of relational outcomes in evaluating the overall success of internal CSR programs. Although prior research has extensively examined mediators between CSR and individual outcomes (e.g., Farooq et al., 2014; Fang et al., 2021; Yan et al., 2021), none of them has considered the mediating mechanism through employees’ perceived CA. By introducing perceived CA as a mediator, our study explores a new underlying mechanism through which employees’ CSR perceptions could influence their relational outcomes. In addition, by qualitatively analyzing employees’ CSR expectations, our findings help to identify significant areas of CSR initiatives that employees are interested in, contributing to a contextualized understanding of Chinese CSR (Ma and Bu, 2021).

This study offers several practical implications. The findings of OCB suggest that for companies, the more they give to various types of CSR activities, the more organizational returns they may receive from their employees. Although the return of CSR may not seem immediately tangible, they will be reflected in employees’ work performance and long-term relationship with the company. In addition, in order to enhance employees’ identification with the company, managers or professionals need to diversify CSR beneficiaries when communicating the company’s CSR to their employees, highlighting how the company’s CSR initiatives consider different stakeholders, including society, communities, employees, and consumers. This study also moves beyond CSR rewards to external relationship management, suggesting that CSR investment can pay off internally. This is particularly important for private companies in China because they are challenged by high mobility and low loyalty of talented employees (Newman et al., 2015). In addition, results of employees’ CSR expectations add descriptive evidence to our understanding of what CSR perspectives companies should pay attention to in a practical manner. From a pedagogical perspective, our study can benefit the business communication curriculum by adding the internal CSR communication perspective to managing employee relationships.

### Limitations and future research

A number of limitations need to be noted regarding the present study. First, this study recruited participants from a single company. Thus, the generalizability of the findings is limited to the context. Future research may use a random, national sample or recruit participants from more representative companies and industries. Second, this study highlighted employees’ overall CSR perceptions without addressing each CSR dimension. Given the diverse CSR interests related to different stakeholders (Turker, 2009b), future research could further explain the effects of specific CSR initiatives. In addition, since researchers have argued that CSR is a context-based concept, to better address the cultural context in China, future research may examine the role of cultural factors. Finally, all measures used in this study were developed from previous research conducted mostly in a Western context. Future research may develop new measures based on the Chinese context.

## Data availability statement

The raw data supporting the conclusions of this article will be made available by the authors, without undue reservation.

## Ethics statement

The studies involving human participants were reviewed and approved by Institutional Review Board of West Texas A&M University. The patients/participants provided their written informed consent to participate in this study.

## Author contributions

YZ contributed to the literature review, method, result, and discussion sections. CD equally contributed to the literature review, method, result, and discussion sections. All authors contributed to the article and approved the submitted version.

## Conflict of interest

The authors declare that the research was conducted in the absence of any commercial or financial relationships that could be construed as a potential conflict of interest.

## Publisher’s note

All claims expressed in this article are solely those of the authors and do not necessarily represent those of their affiliated organizations, or those of the publisher, the editors and the reviewers. Any product that may be evaluated in this article, or claim that may be made by its manufacturer, is not guaranteed or endorsed by the publisher.

## Appendix A

Constructs and measures.

**Table:**

Construct	Measures	Mean (SD)
Employee CSR perceptions	Makes investment to create a better life for future generations	4.85 (1.50)
	Supports NGOs working in problematic areas	4.74 (1.46)
	Contributes to campaigns and projects that promote society’s well-being	5.03 (1.33)
	Encourages employees to develop their skills and careers	5.21 (1.50)
	Is primarily concerned with employees’ needs and wants	4.56 (1.53)
	Supports employees who want to acquire additional education	5.13 (1.47)
	Respects consumer rights beyond the legal requirements	5.04 (1.03)
	Provides full and accurate information about products to customers	5.48 (1.36)
	Considers customer satisfaction as highly important	5.60 (1.34)ƒ
	Always pays taxes on a regular and continuing basis	6.08 (1.26)
	Complies with legal regulations completely and promptly	6.07 (1.26)
Employee-organization identification	I feel strong ties with my company	5.34 (1.29)
	I experience a strong sense of belongingness to my company	5.30 (1.37)
	I am part of my company	5.50 (1.18)
Corporate ability	I associate this company with innovative products	5.52 (1.30)
	I associate this company with market leadership	5.50 (1.22)
	I associate this company with good quality products	5.44 (1.27)
	I associate this company with efficient manufacturing facilities	5.25 (1.36)
	I associate this company with expertise in manufacturing products	5.29 (1.44)
	I associate this company with global success	4.55 (1.56)
Employee-organization relationships (EOR)	This organization treats people like me fairly and justly	4.93 (1.70)
	Whenever this organization makes an important decision, I know it will be concerned about people like me	4.68 (1.60)
	This organization can be relied on to keep its promises	5.27 (1.46)
	I believe that this organization takes the opinions of people like me into account when making decisions	4.81 (1.57)
	I feel very confident about this organization’s skills	4.97 (1.57)
	This organization has the ability to accomplish what it says it will do	5.15 (1.53)
	I am happy with this organization	5.10 (1.50)
	Both the organization and people like me benefit from the relationship	5.11 (1.51)
	Most people like me are happy about their interactions with this organization	4.95 (1.48)
	Generally speaking, I am pleased with the relationship this organization has established with people like me	5.10 (1.42)
	I feel that this organization is trying to maintain a long-term commitment to people like me	5.14 (1.43)
	I can see that this organization wants to maintain a relationship with people like me	5.24 (1.41)
	There is a long-lasting bond between this organization and people like me	5.07 (1.44)
	Compared with other organizations, I value my relationship with this organization more	5.18 (1.38)
Organizational citizenship behavior (OCB)	Attend functions that are not required but that help the organizational image	5.66 (1.20)
	Keep up with developments in the organization	5.56 (1.29)
	Defend the organization when other employees criticize it	5.47 (1.25)
	Offer ideas to improve the functioning of the organization	5.14 (1.31)
	I would like to recommend the company as a good employer	5.28 (1.49)
